# Advancing the protection of marine life through genomics

**DOI:** 10.1371/journal.pbio.3001801

**Published:** 2022-10-17

**Authors:** Madeleine J. H. van Oppen, Melinda A. Coleman

**Affiliations:** 1 Australian Institute of Marine Science, Townsville, Queensland, Australia; 2 School of BioSciences, The University of Melbourne, Parkville, Victoria, Australia; 3 Department of Primary Industries, NSW Fisheries, National Marine Science Centre, Coffs Harbour, New South Wales, Australia; Smithsonian Institution, UNITED STATES

## Abstract

The rapid growth in genomic techniques provides the potential to transform how we protect, manage, and conserve marine life. Further, solutions to boost the resilience of marine species to climate change and other disturbances that characterize the Anthropocene require transformative approaches, made more effective if guided by genomic data. Although genetic techniques have been employed in marine conservation for decades and the availability of genomic data is rapidly expanding, widespread application still lags behind other data types. This Essay reviews how genetics and genomics have been utilized in management initiatives for ocean conservation and restoration, highlights success stories, and presents a pathway forward to enhance the uptake of genomic data for protecting our oceans.

## Abstract 2

The rapid growth of genomic techniques is transforming how we protect, manage, and conserve marine life. In this Essay, Madeleine van Oppen and Melinda Coleman explore how genomic insights can be used and operationalized to conserve marine life in the Anthropocene.

## The need for genomic data

No part of our oceans is left untouched by humans, with many marine species and habitats showing severe declines in health and abundance as a consequence of anthropogenic disturbances [1]. Important ecosystems such as coral reefs, seagrass meadows, and kelp forests are declining from the direct and indirect effects of climate-driven warming, more severe and frequent extreme events, and disease outbreaks [2–5]. Many marine animals have experienced severe population bottlenecks from overharvesting [6]. This global trend of biodiversity and ecosystem declines [7] has prompted a range of conservation measures to combat loss and protect the valuable goods and services provided by species and ecosystems. Progress towards removing local stressors and reversing extant species and habitat loss is accelerating with 2021 heralding the UN decade of ocean science. Measures to protect against rapid climate change are perhaps most challenging [6] because of the global nature of this stressor and the need for novel interventions that are only just being developed.

Conservation efforts are most successful when they are underpinned by scientific data that inform, for example, which species, populations, or places are most vulnerable and require protection. Scientific data are also crucial in measuring success of conservation efforts and in designing management interventions to reverse degradation and loss. Data types traditionally used to inform conservation planning and decision-making are diverse and include: species and habitat distribution and abundance maps; degree of disturbance; threats and risks; characteristics of the physical and chemical environment such as temperature, salinity, substratum type, and the movement of ocean currents; and human usage of the marine environment. The application of genomic data in marine conservation is also gaining traction, but still lags behind these other data types [8]. Yet, genomic approaches hold huge promise in advancing marine conservation and restoration; they provide certain insights that cannot be garnered from any other data source and often give more power to address important and new questions. Moreover, conserving and managing marine systems under climate change will require novel genomic interventions to ensure species and ecosystem persistence, making this data source critical to future conservation efforts.

The term “genetics” refers to the study of a subset of genes or other parts of the genome, while we define “genomics” as genome-wide studies or the use of reference genomes and high-throughput genomic techniques (see Box 1 for a glossary of terms used in this article). Most characteristics of a living being are encoded in its genome, and thus, genomic information lies at the basis of an organism’s appearance, behavior, and physiology. In the context of conservation and restoration, genetic and genomic approaches can be used to guide and enhance traditional conservation actions as well as to design more recent assisted evolution approaches [9]. Importantly, genomic approaches are essential components of most biotechnological manipulations aimed at the development of genome-edited and genetically modified organisms (GMOs) or synthetic life forms that benefit or relieve pressures on marine life [10]. In this Essay, we review conservation insights and interventions that rely on or can be greatly enhanced by genetics and genomics approaches and discuss how these approaches can progress marine conservation and restoration efforts. We then explore some stand-out examples of where genetic and genomic data have been operationalized and make recommendations on how to continue to expand the use of genomics to advance marine conservation and restoration.

**Box 1. Glossary**

**Acclimatization**

The physiological adjustment of an organism to a change in its environment within its lifetime and via non-genetic processes.

**Adaptation**

The change in allele frequencies in a population across generations in response to a selective force, leading to a shift in fitness.

**Adaptive management**

An ongoing, iterative process for management and monitoring that may be adjusted over time as our understanding of an ecological system’s response to management improves.

**Assisted evolution**

The acceleration of naturally occurring evolutionary processes to enhance certain traits.

**Assisted gene flow**

The managed movement of individuals with favorable traits (alleles/genotypes) into populations (unidirectional) to reduce local maladaptation to climate or other environmental change.

**Biobank**

A repository that stores biological samples.

**Effective population size (*N*_*e*_)**

The number of breeding individuals in a population; *N*_*e*_ determines the rate of change in the composition of a population caused by genetic drift, which is the random sampling of genetic variants in a finite population.

**Gene flow**

The transfer of genetic material between populations via immigration of individuals and subsequent interbreeding of immigrants with the native populations.

**Gene drive**

A phenomenon whereby a particular heritable element biases inheritance in its favor, resulting in the gene becoming more prevalent in the population over successive generations.

**Genetic diversity**

An estimate of the number gene variants of a subset of genes within a population or species.

**Genetic engineering (also called genetic modification)**

The manipulation of an organism’s genes by introducing, eliminating, or rearranging specific genes using the methods of modern molecular biology, particularly those techniques referred to as recombinant DNA technologies (i.e., technologies using enzymes to cut and paste together DNA sequences of interest).

**Genetically modified organism (GMO)**

An organism that has been modified using gene technology or an organisms that has inherited modified traits from a GMO.

**Genome editing (also called gene editing)**

The targeting of functional proteins to precise locations in the genome to modify the coding sequence or activity of genes.

**Inbreeding**

Mating between close relatives.

**Introgression**

The transfer of genetic material from one species to another or between divergent populations of the same species.

**Managed breeding**

The controlled breeding of organisms to maximize genetic diversity and/or environmental tolerance and fitness of offspring.

**Probiotics**

Live microorganisms that are intended to have health benefits when consumed or applied to the body.

**Reference genome**

Contiguous and accurate genome assembly representative of a species in which the coordinates of genes and other important features are annotated.

**Synthetic biology**

A growing discipline that involves the application of engineering principles to biology; it aims to redesign and fabricate biological components and systems that do not already exist in nature.

**Transgenic organism**

An organism in which a foreign gene or non-coding DNA fragment is artificially introduced and stably integrated in its genome

## Genetic and genomic diversity data to inform marine conservation and restoration interventions

**Fig 1:**
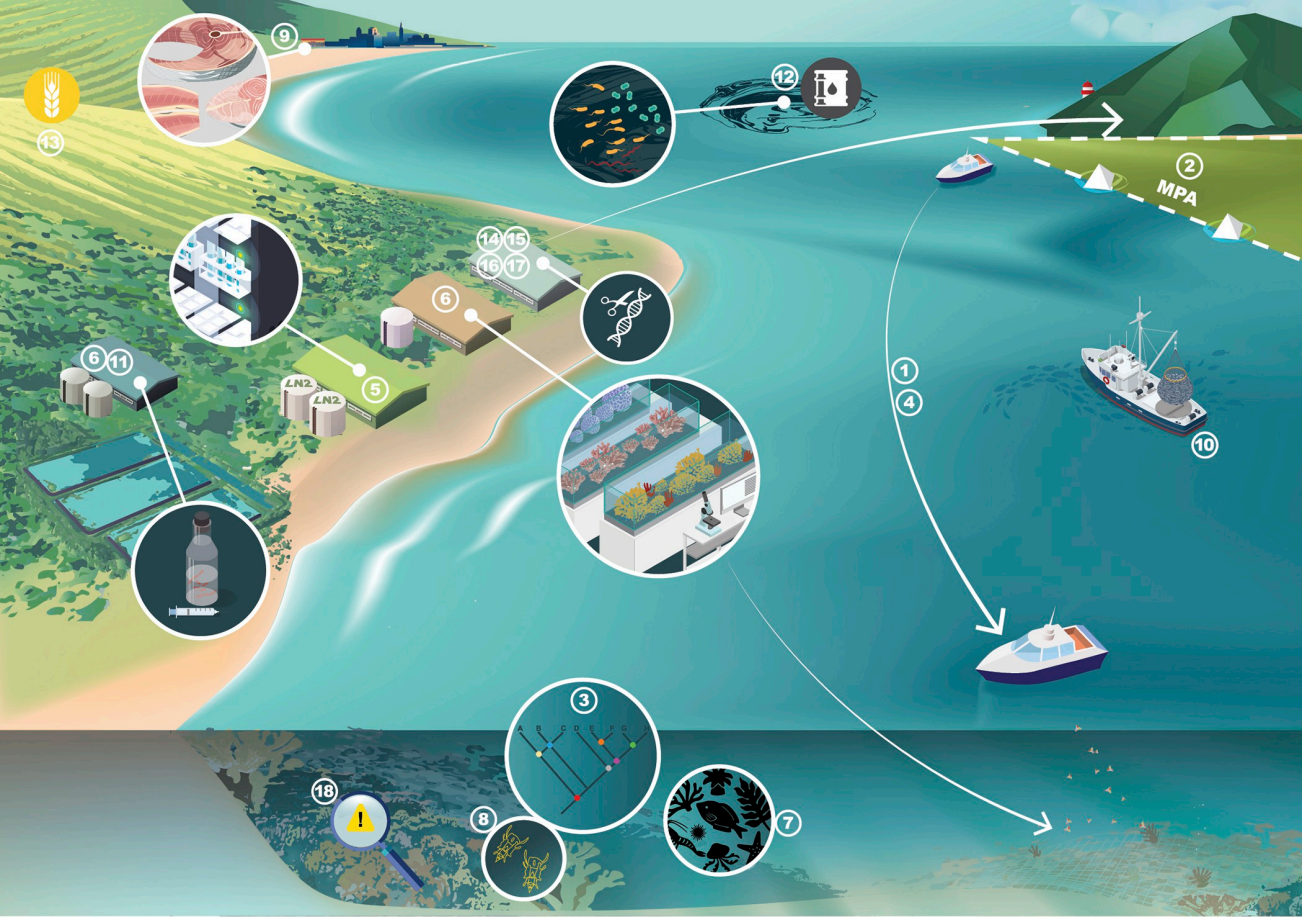
Infrastructure for genomic/genetic marine conservation actions and interventions. Cartoon depicting the conservation actions and interventions addressed in this Essay and showing the major infrastructure required for each. Numbers relate to actions/interventions in Table 1.

Genomics provides information on variation at parts of the genome (i.e., loci) that have no effect on fitness or adaptation (loci that are selectively neutral) and those that do (functional loci). The most basic parameter provided by genomics is an estimate of genetic diversity. The genetic diversity of a population shows a positive correlation with its adaptive potential and fitness, and extreme loss of genetic diversity (genetic erosion) can lead to and be a consequence of inbreeding, a decline in fitness, and an increased risk of extinction [11]. Knowledge of genetic erosion can contribute and motivate the assessment of the conservation status of threatened or endangered species, e.g., for classification under the IUCN Red List framework, as well as for developing management responses (Table 1 and Fig 1 (action 1)) [12] (but see [13] on a cautionary note of using genetic diversity information at neutral loci only). Neutral loci are useful for estimating gene flow among populations (i.e., connectivity), introgression between species, and effective population sizes. They can also be examined to spatially map parents and their offspring as a measure of dispersal distances and directionality, to unveil spatial genetic structure (Table 1 and Fig 1 (action 2)), and to delineate species boundaries and resolve taxonomic uncertainties (Table 1 and Fig 1 (action 3)). Functional parts of the genome that are under natural selection can be interrogated to assess whether adaptation to the local environment has occurred and how adaptive genetic variants are partitioned across the distribution range of a species. Data on neutral and adaptive genetic variation may inform threatened species recovery plans (Table 1 and Fig 1 (action 1)) or enhance the design of marine protected area networks (Table 1 and Fig 1 (action 2)) [14], for example, by assisting in the decision-making process to restrict extractive activities or stressors in particular populations because they harbor unique genetic variants or are genetically depauperate, or by including knowledge on the extent and direction of gene flow. Such data are also important to designing restoration programs so that donor individuals for transplantation or seeding can be chosen to replicate or boost natural levels of genetic diversity and structure (Table 1 and Fig 1 (action 4)) [15]. Biobanking, assisted gene flow, and managed breeding efforts (Table 1 and Fig 1 (actions 4–6)) [16] will benefit from genomic data by identifying genetically distinct individuals or individuals carrying adaptive alleles. Finally, genomic data are important in measuring success of conservation and restoration interventions and assessing any impacts on natural populations.

**Table 1:** Genomic/genetic marine conservation actions and interventions addressed in this Essay.

	Conservation actions and interventions	Genomic tools and data types	Adaptive or neutral genetic variation	Technological readiness	Level of current uptake
1	Genetic rescue (translocation)	SNPs, RG, GS	N	H	L
2	Marine protected area design and spatial planning	eDNA, MB, msats, SNPs, RG, GS	A, N	H	M
3	Species identification and delineation	eDNA, mtDNA, GS	N	H	H
4	Assisted gene flow (translocation) and restoration design (provenance)	SNPs, RG, GS, GWAS	A, N	H	L
5	Biobanking	SNPs, RG, GS, GWAS	A, N	H	L
6	Assisted evolution (via managed breeding)	SNPs, RG, GS, GWAS	A	H	L
7	Biodiversity monitoring	eDNA, MB, mtDNA, msats, SNPs, GS, MG	N	H	M
8	Early warning biomarkers of invasives and pests	eDNA, MB	N	H	L
9	Combating illegal fishing and mislabeling	eDNA, MB, mtDNA, SNPs, GS	A, N	H	M
10	Managing fisheries	msats, mtDNA, SNPs, GS	A, N	H	M
11	Microbiome manipulation	RG, MB, MG	A	M	L
12	Microbial bioremediation	RG, MG	A	M	L
13	Alleviating marine stressors ex situ	RG, GEd, GE, Syn Bio	A	L	L
14	Provisioning of marine life services ex situ	RG, GEd, GE, Syn Bio	A	L	L
15	Evolutionary rescue via genome editing	RG, GS, Ged, GWAS	A	L	L
16	Pest control	RG, GE, Ged, Syn bio, GD	A	L	L
17	De-extinction	RG, GEd	A	L	L
18	Genomic vulnerability analyses	SNPs, RG, GS, GWAS	A	H	L

Numbers link these activities to the text and Fig 1.

Rankings are high (H), medium (M), and low (L).

A, adaptive; eDNA, environmental DNA; GD, gene drives; GE, genetic engineering; GEd, genome editing; GS, genome sequencing (including whole-genome sequencing, reduced complexity, and shallow genome sequencing); GWAS, genome-wide association studies; MB, metabarcoding; MG, metagenomics; msats, microsatellites; mtDNA, mitochondrial DNA; N, neutral; RG, reference genome; SNPs, single-nucleotide polymorphisms; Syn Bio, synthetic biology.

## Biodiversity and invasive/pest species monitoring with environmental DNA

Monitoring the status of marine biodiversity with traditional methods such as SCUBA-based surveys or plankton tows is a resource- and time-intensive undertaking, and species-level assessments are virtually impossible from in-field surveys or imagery. Moreover, rare or cryptic species are often missed in traditional surveys and many parts of our ocean, such as the deep sea or turbid areas, are difficult to survey visually. The analysis of environmental DNA (eDNA) is emerging as a more feasible alternative or complement to traditional visual diversity surveys (Table 1 and Fig 1 (action 7)) [17] and techniques are improving for assessments of relative biomass or abundance [18]. Deep sequencing of a particular DNA region (DNA metabarcoding) of all the DNA within an environmental sample (e.g., water, sediment, feces, or organismal tissues) has been extensively used to assess diversity of prokaryotes (bacteria and archaea; mostly targeting the 16S ribosomal RNA gene) as most environmental samples contain many prokaryotic cells. There are no non-genomic methods that can replace this approach due to the small number of morphological traits available to distinguish among prokaryote taxa and the vast number of undescribed marine prokaryote species. Similarly, eukaryotic microbial communities are commonly characterized from water samples via this approach (by targeting 18S or other eukaryotic genes). Importantly, most environmental samples not only contain microbial cells, but also contain a diverse pool of DNA shed by eukaryotic and prokaryotic life that can also be characterized with DNA metabarcoding methods. Linking DNA barcodes to taxonomic descriptions of species allows for eDNA analysis to provide species lists, and assessments of genetic diversity and taxon abundance are possible under some circumstances [19]. While marine eDNA is a novel field, its potential to provide spatial and temporal data sets much larger and with improved resolution compared to those that can be acquired with traditional methods is enormous [20]. Methods other than metabarcoding have been successfully applied, such as quantitative or droplet digital PCR. Further, with the ever-declining cost of high-throughput DNA sequencing, obtaining metagenomes rather than metabarcodes from eDNA will become achievable in the near future, expanding eDNA analysis to yield data on the functional potential of marine communities.

Biological invasions and pest outbreaks are another biodiversity threat of great concern across the marine realm where eDNA can play an important role by assessing their occurrence, spread, and biomass (Table 1 and Fig 1 (action 8)) [21]. For example, a highly sensitive nucleic acid lateral flow device targeting the mitochondrial DNA (mtDNA) *COI* gene was able to detect the coral-eating crown-of-thorns sea star from eDNA in seawater in low density (non-outbreak) populations [22]. Such genetic information allows early intervention and may be used to direct pest management measures to the areas where these are most needed.

## Forensic genomics

Combatting illegal trade is important for supporting sustainable use and protection of harvestable marine resources and to ensure the traceability of produces from source to plate. It is also vital for conservation of threatened species where harvesting is often a key threatening process. Morphological identification of harvested marine species is often challenging, inaccurate, and sometimes impossible, and genomic techniques present a significant advance in achieving these tasks (Table 1 and Fig 1 (action 9)). To date however, the use of genomic techniques in marine forensics has only been sporadic [23] but it is often pivotal in prosecuting illegal activities.

Identifying seafood products to species level is often done through mtDNA barcoding because it is impossible to morphologically identify species from flesh or body parts alone (e.g., fish fillets, caviar). This technique is particularly valuable when applied to illegal harvest of threatened species [24]. Genomic techniques are also used to identify the population of origin of seafood products when they are suspected to have been harvested illegally, but this task is challenging for many species for which no baseline of spatial genetic structure exists [25]. For high-value species such as salmon, cod, herring, and hake, assignment tests based on microsatellite or single-nucleotide polymorphism (SNP) data have been used to distinguish individuals from different areas for compliance purposes [26] and to identify genetic pollution from aquaculture farms [27]. For many species, a baseline of genetic or genomic differentiation must be obtained, against which target samples can be compared. This can be done at a spatially coarse level using less variable mtDNA haplotypes [24]. For many species, however, a finer genomic resolution is required (genetic fingerprinting using microsatellite markers, SNPs, or whole-genome sequencing) to determine where a harvested resource has come from. Such an approach might be necessary, for example, to identify if species have been illegally harvested from areas closed to fishing, but for many species (e.g., lower value species), the cost and time involved in such an approach may preclude its practical use. More recently, metagenomic sequencing of product microbiomes has also been shown to be useful in tracing the source of seafood.

Forensic genomics has recently advanced to allow identification of the cause of death and mass mortality in natural populations where the stressor may not be obvious or has abated prior to mortality [28]. Such an approach is dependent on identifying signatures of selection in survivors via outlier SNPs and their function to pinpoint likely stressors. This method, however, relies heavily on populations containing sufficient a priori genetic diversity upon which selection can act, as well as genomic data or samples from before the event or in an unaffected area and a good reference genome to map SNPs to gene functions. Nevertheless, this approach allows us to understand the stressors responsible for population decline and manage accordingly into the future.

## Managing fisheries

Conserving harvested marine species is paramount to ensure their long-term sustainable use and health. Fisheries scientists must be able to accurately identify fish stocks or units, monitor population sizes, and understand movements and migrations to protect key areas to inform management policies. Such information is necessary to assess the status of fished stocks and for managers to design appropriate strategies such as setting fishing quotas and spatiotemporal fishing closures. Traditional methods to achieve such information include mark-recapture studies, otolith chemistry, fishery-independent surveys, egg or larval collection, and long-term catch and effort data, but the accuracy and spatial resolution of data can be greatly advanced with genetic and genomic techniques (Table 1 and Fig 1 (action 10)) [29,30].

Stock assessments can be improved by understanding the identity and spatial extent or boundaries of fish stocks. Genetic markers such as mtDNA, allozymes, and microsatellites have been used for decades to achieve this [29]. Such data have revealed cryptic species, hybridization among stocks, and spatial and temporal genetic structure that can be considered by fisheries managers when assessing stock structure and setting appropriate harvesting levels. The advent of high-throughput sequencing has opened the door for fisheries managers to also consider the adaptive structure of fish stocks. For well-studied species such as hake and cod, analyses of SNPs under selection have revealed significant additional structure that was not present in neutral markers alone [31]. Genotype-environment analyses can also reveal adaptive structure that has implications for harvesting and restocking programs as well as understanding the response of fisheries to climate change. Moreover, studies of adaptive genetic diversity can allow fisheries managers to assess the impact of fishing on fish stocks or restocking on natural populations. Changes in adaptive genetic diversity over an 80-year period of over-harvesting were demonstrated in Atlantic cod with fisheries-induced selection associated with life history suggested to be partly responsible [32].

Estimating population size is a key yet difficult component of fisheries management and has traditionally been done via modelling, mark recapture studies, egg or larval surveys, or catch per unit effort (CPUE). Low-coverage genomic data (e.g., RADSeq) can be used to estimate effective population size (*N*_*e*_). An alternative approach to inferring population size is through kinship of individuals caught [33]. This technique relies on identifying parent–offspring pairs or full and half siblings using SNP or microsatellite genotyping of adults and juveniles [34]. It has been used successfully for managing southern bluefin tuna, with kinship analyses suggesting that the stock was less depleted and more productive than indicated by traditional CPUE methods [35].

## Microbially mediated assisted evolution

Host-associated microorganisms perform functions that can be beneficial to their host and such microbes may be harnessed as probiotics (Table 1 and Fig 1 (action 11)) [36]. Genomic information on the genes and metabolic pathways contained within microbial genomes is of great value for selecting potential probiotics as this points to their functional potential, although phenotypic data can also be used for strain selection. Bacterial or fungal probiotics are commonly used to improve gut health in humans; to increase growth, disease resistance, and overall health in aquaculture species; or to enhance growth and environmental stress tolerance in crop species. Less frequently, probiotics have been implemented as wildlife medicine [37].

Reference genomes are required to guide artificial selection (i.e., directed or experimental evolution) of microorganisms that may change trait values to boost environmental stress or disease tolerance of their hosts once these enhanced microbes are reintroduced into the host. The dinoflagellate photosymbionts of corals, for example, show increased in vitro thermal tolerance after long-term thermal selection which is sometimes transferrable to the coral host animal [38]. Similarly, host-associated bacteria and fungi that are culturable can be evolved outside the host in the laboratory [39]. This approach has been demonstrated for a number of bacterial and fungal taxa, but to our knowledge has not yet been applied to marine conservation. While changes in trait values in response to artificial selection can be assessed phenotypically, high-quality reference genomes that provide knowledge on the genomic mutations responsible for phenotypic changes will assist in the identification of naturally occurring beneficial microbes and the adaptive gene variants they harbor and can also direct genetic engineering efforts.

## Provisioning of services and reducing pressures by natural, genetically modified, and synthetic organisms

Microbial life is highly diverse, and microbes have many traits that can be employed to alleviate or remove some of the detrimental impacts of human activities on marine ecosystems (Table 1 and Fig 1 (action 12)). Such traits are often identified from reference genomes, from which gene function and metabolic pathways, and thus functional potential, can be derived. One example is the microbial degradation of pollutants. For example, some bacteria, microalgae, and fungi can break down hydrocarbons and could assist in the mitigation of marine oil spills [40]. Another example is the challenge in removing the vast amounts of plastics that are accumulating in marine habitats and organisms. After entering the sea, plastics are rapidly colonized by microbes, with some bacteria and fungi possessing the capability to degrade plastics. This provides a huge potential for microbial bioremediation of plastic pollution, although complete mineralization has yet to be demonstrated outside the laboratory [40].

Conservation actions that benefit marine life by limiting or removing stressors can also be applied ex situ (Table 1 and Fig 1 (action 13)). Genomics is playing a large role in development of innovative solutions to environmental problems on land that have direct or indirect benefits to the marine environment. For example, genomic analyses can identify bacteria that can be employed to reduce emissions of the greenhouse gas methane from agriculture (e.g., rice paddies, ruminants, and meat production form non-ruminants) [41], as such positively influencing marine ecosystems by slowing down climate warming. Similarly, bacteria are critical components of the wastewater treatment process and play important roles in reducing nutrients and chemicals that enter the sea. Genomic monitoring of the structure and potential function of microbial communities in wastewater treatment plants is critical to ensure optimal efficiency of the system [42].

Limiting agricultural runoff of nutrients, sediments, and agrichemicals via development of genetically engineered crops that require less fertilizer and water [43] will have indirect benefits for the marine environment by reducing pressures that come from catchments (Table 1 and Fig 1 (action 13)) [44]. Furthermore, the use of synthetic biology to develop more sensitive and cheap biological sensors (i.e., biologically encoded elements designed to react to a level of a chemical, metal, or analyte) could detect when thresholds of stressors (e.g., metal pollution, nutrient enrichment, or oxygen depletion) are being approached in situ and trigger prompt management actions that seek to limit those stressors prior to any impact occurring. Synthetic biology also holds promise for developing alternatives to marine bioproducts that may remove or limit harvesting pressures on marine species (Table 1 and Fig 1 (action 14)). Horseshoe crabs and shorebird predators that rely on their eggs are in global decline due to the unsustainable harvesting of horseshoe crabs for biomedical testing of bacterial endotoxin activity in the manufacturing of vaccines, medications, and certain medical devices. A synthetic alternative (recombinant rFC) to the horseshoe crab blood was developed in 1997, which has recently been shown to have comparable performance to the wild-harvested product. Pharmaceutical manufacturers may thus be able to reduce the cost and time required for testing by switching to the rFC assay [45], and this will severely relief pressures on horseshoe crabs [46]. A similar example is the synthetic production of triterpene squalene, a chemical first described from the liver of the deep-sea shark, *Squalus* spp., which supports a huge commercial market as a food supplement, cosmetic, and pharmaceutical. While some plants, fungi, and other microbes can also synthesize squalene, it has been challenging to upscale squalene production from natural sources other than shark. Fortunately, synthetic biology approaches are currently being explored to make the production of this valuable compound by microorganisms commercially viable [47], which will remove the need to fish sharks for this purpose.

## Evolutionary rescue and biocontrol with genome editing and genetic engineering technologies

The genetic adaptation that allows population recovery from environmentally induced demographic effects that otherwise would have caused extinction is known as evolutionary rescue. While evolutionary rescue can occur naturally, management and conservation actions may assist the evolutionary rescue process. Such actions may be currently controversial, but it is vital that the genomic information and science underpinning such strategies are advanced to enable sensible use when the time comes. Evolutionary rescue can be achieved by assisted gene flow (Table 1 and Fig 1 (action 4)) [48] or managed breeding (Table 1 and Fig 1 (action 6)) or alternatively via genome editing (Table 1 and Fig 1 (action 15)). Genome editing requires detailed understanding of allelic variants underpinning phenotypic traits as specific loci need to be targeted with high precision and the nucleotide substitutions required to create the better adapted alleles need to be known. Whole-genome association studies are a powerful approach to obtaining such information. One major challenge of evolutionary rescue via genome editing, however, is the generally multigenic nature of stress tolerance traits [49], but targeting transcription factors may overcome this issue to some extent [50].

The insertion, knock-out, or overexpression of genes are other powerful conservation applications of genome editing as well as earlier transgenic methods. A much-cited success story from the terrestrial realm is that of the American chestnut that has been devastated by a fungal disease (blight). Insertion of a wheat oxalate oxidase gene significantly increases blight resistance that is heritable and this represents a major step towards restoration of these once dominant trees [51]. We are not aware of any marine examples relevant to conservation, but transgenic fish, mollusks, micro- and macro-algae, and sea urchins have been successfully developed for other ex situ purposes [52]. For example, to better understand acclimatory responses, the insertion and expression of the carp muscle form III of creatine kinase gene into the zebrafish genome allowed the transgenic fish to swim at low temperatures while the wild-type fish could not [53]. Knock-outs or overexpression of a gene may also enhance phenotypic traits, such as stress tolerance [54,55]. These emerging demonstrations of the tractability of gene editing in marine species provide important scientific knowledge and insight that may one day be used in natural systems.

A rather controversial application of genome editing is the use of gene drives to eradicate invasive pest species (Table 1 and Fig 1 (action 16)) [56,57]. Gene drives rely on spread via sexual reproduction; therefore, their application is mostly relevant to species with short generation times. Gene drives are an emerging but controversial tool and have rarely been applied due to concerns about containment, environmental risks, and ethics. Future research and development need to solve these concerns before this approach may become acceptable for (marine) conservation [58]. For example, use of gene drives to eradicate marine pests in isolated areas such as remote islands where containment is ensured may be feasible. Moreover, incorporation of additional synthetic elements that provide barriers to downstream sexual reproduction could limit propagation of gene drives to population, species, or areas of interest [59].

## De-extinction

De-extinction or resurrection biology is the process of generating an organism that resembles an extinct species. This can be achieved via selective breeding (which does not require genomic information), cloning, or genome editing (Table 1 and Fig 1 (action 17)). While there are no marine examples, this is being explored for terrestrial species. For example, the extinct quagga, a subspecies of the plains zebra, is currently being resurrected via selective breeding [60]. De-extinction can also be achieved via cloning, which involves the extraction of the nucleus containing the chromosomes from a preserved cell of the extinct species and inserting it into an egg (from which the nucleus has been removed) of a closely related species. This method was used to resurrect the extinct Pyrenean ibex to produce 1 animal that unfortunately only survived for a few minutes. Finally, genome editing can be used to change the DNA sequence of a close relative of the extinct species to that of the extinct species, and this is one of the approaches being applied to recreate the woolly mammoth from elephants where approximately 60 elephant genes will be edited into the woolly mammoth counterpart [61]. Detailed knowledge of the genome sequence of both the extinct species and the close relative is required for this process.

Should de-extinction be considered for marine species? At least 20 marine species are known to have recently disappeared from the world’s oceans [62], including the great auk which is being considered for de-extinction. Some people argue that funds spent on de-extinction would have a much greater conservation impact if directed at preserving species that are threatened but still alive [63]. Spending large sums of money on de-extinction can perhaps be justified for extinct keystone species that are critical to support an ecosystem or are the sole habitat builder of a particular ecosystem. Further, the technological advancements that are being developed through de-extinction science are important to ensure readiness to tackle future problems and because of the serendipitous findings that often accompany such developments.

Instead of resurrecting a species, in some instances the DNA of threatened species may be preserved via interspecific hybridization. This process may preserve genes and gene variants from going extinct even if the initial carrier of this genomic information goes extinct. This preserved genetic variation may persist in the hybrid and even integrate into purebred species via back-crossing and may provide novel traits and increase stress and disease tolerance [64]. Indeed, hybrid vigor or heterosis can increase thermal tolerance in corals [65] and kelps [66] and could be used to facilitate adaptation to climate change.

## The future of marine conservation in the genomics age

There are many technological advances and instances of successful use of genomic data in marine conservation, but its uptake is far from widespread even with positivity surrounding its value [66]. Barriers to the widespread uptake of genomic data include its relatively recent availability, rapid trajectory of advancement, cost, analytical barriers, and social and communication aspects [67]. Increasing uptake will require development of better analytical pipelines and computational resources. However, increasing uptake in on-ground conservation actions will require collaborative partnerships between managers and scientists and the cooperative development of accessible information sharing platforms.

### Tools and collaborations

**Fig 2:**
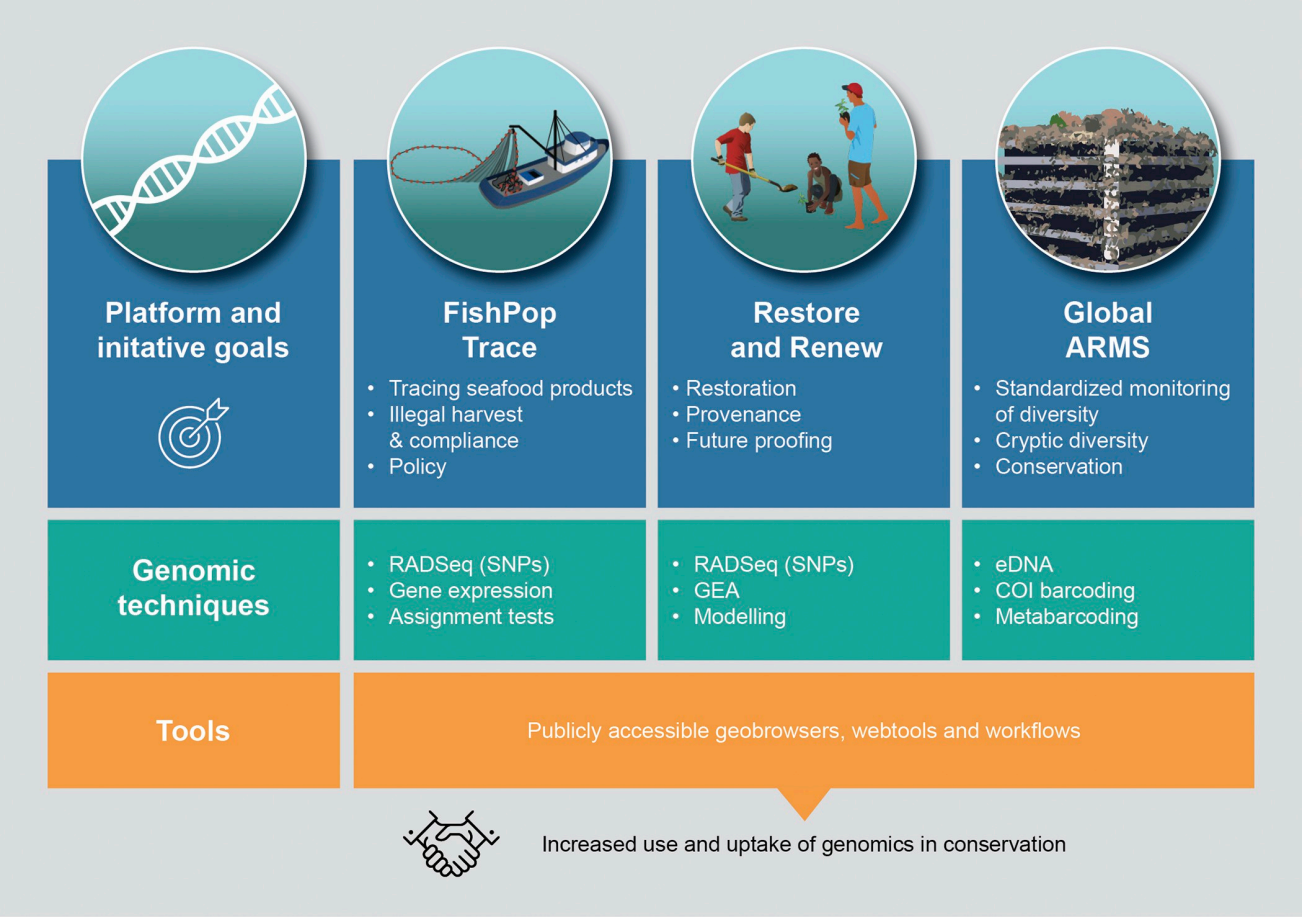
Tools, techniques, and platforms for using genomic data in marine conservation. Examples of applied online tools and platforms that assist biodiversity managers and conservation practitioners in the use genomic data. GEA; genotype-environment associations.

There are some excellent examples of genomic data being operationalized for conservation through applied online tools and platforms that are accessible to managers and practitioners (Fig 2). Importantly, some of these tools negate the need for in depth understanding and collection of the underlying genomic data and techniques, instead translating complex genomic data and concepts into applied management solutions. For example, the FishPopTrace project utilizes genome-wide technologies to genotype SNP markers in commercially important fish to allow identification of stocks and trace the geographical origin of fish to identify illegal trade and mislabeling [68]. The UK government and the Marine Stewardship Council use this stock traceability information in a regulatory and authenticity verification framework. Another example is the Restore and Renew initiative that responds to the needs for restoration practitioners and community groups to develop climate resilient restoration practices [69]. The program combines genotype-environment associations in native plant species with climate modelling to determine appropriate provenance of seeds to match either extant or future climatic conditions. A user-friendly webtool allows managers to select various climate scenarios and time periods for which to restore to and produces maps showing where seed could be collected to match those conditions, negating the need for non-academics to grapple with genomic data. With marine restoration set to accelerate as a means to combat degradation, similar tools are needed to guide restoration efforts, particularly for foundation species such as corals, seagrasses, and kelps that underpin entire ecosystems. The global and European ARMS (Autonomous Reef Monitoring Structures) programs that combine standardized benthic settlement structures with new eDNA barcoding technologies to characterize biodiversity and monitor change (Fig 2) provide a good example. These are particularly valuable in assessing cryptic species or those that are morphologically difficult to identity and the standard sampling strategy makes global comparison of ocean health possible. Online workflows to facilitate accessibility of data are being developed.

### Proactive management based on genomics

We can also transform management and conservation from being reactive to proactive by embracing new genomic analyses that have potential to forecast and anticipate future adaptability of marine species to climate change (Table 1 and Fig 1 (action 18)). Modelling relationships between genomic diversity and current versus future environmental conditions allows the unique opportunity to forecast where there may be mismatch between future ocean conditions and a species’ ability to adapt, providing the opportunity for early and proactive interventions. These new analyses are beginning to gain traction in terrestrial settings, but have only recently been applied to key marine habitats that underpin entire ecosystems (e.g., kelp forests [70]) revealing a likely inability of these species to keep pace with climate change. Predictive genomic vulnerability assessments will be vital for harvested species and key habitat formers to enable proactive adaptive management under climate change.

## Conclusions

Marine biodiversity is rapidly declining, and many marine ecosystems are under threat from anthropogenic disturbances including climate change. As such, there is an urgent need for genomic information to be incorporated in resource management actions for marine ecosystems and the foundation species that underpin them. This will require a commitment to long-term genomic monitoring that is coupled with ecological metadata to assess species and ecosystem vulnerability and to allow adaptive management. Further, investment in broadening and enhancing genomic resources, such as reference genomes, is needed to understand organismal responses to climate change and to pave the way for transformative solutions. Enabling access and use of genomic information by conservation planners and managers will require the development of suitable online platforms and enhanced collaboration between the various stakeholders of marine ecosystems. We encourage marine conservation genomicists to go beyond publishing their results in the scientific literature and direct their efforts towards such initiatives, and we call on funding agencies to invest in the development of accessible platforms that operationalize genomic data. Genomic intelligence has the potential to considerably improve conservation and restoration programs, and thus it is critical that the gap between genomics experts and marine biodiversity managers is bridged.

## Abbreviations

ARMS: Autonomous Reef Monitoring Structures
CPUE: catch per unit effort
eDNA: environmental DNA
GMO: genetically modified organism
mtDNA: mitochondrial DNA
SNP: single-nucleotide polymorphism
